# First-episode olfactory hallucination in a patient with anxiety disorder: A case report

**DOI:** 10.3389/fpsyt.2022.990341

**Published:** 2022-09-20

**Authors:** Xingmei Jiang, Yiwen Yuan, Zhixiong Li, Ying Ou, Zhe Li

**Affiliations:** ^1^Mental Health Center, West China Hospital, Sichuan University, Chengdu, China; ^2^Sichuan Clinical Medical Research Center for Mental Disorders, Chengdu, China; ^3^The Third Department of Clinical Psychology, Karamay Municipal People’s Hospital, Karamay, China

**Keywords:** anxiety disorder, anti-anxiety treatment, case report, first-episode, olfactory hallucination

## Abstract

**Background:**

Olfactory hallucination refers to olfactory perception in the absence of chemical stimuli. Although it has been associated with many neurological and psychotic disorders, it has rarely been reported as the first and only symptom in patients with anxiety disorder, and its treatment remains inadequate.

**Case summary:**

A 66-year-old woman who had been experiencing gradually worsening olfactory hallucinations for almost 4 years was diagnosed with generalized anxiety disorder. Olfactory hallucination disappeared after treatment with anti-anxiety drugs.

**Conclusion:**

Olfactory hallucination can be the first and only symptom in patients with anxiety disorder and may be effectively treated with anti-anxiety medication. In fact, it can precede the diagnosis of anxiety disorder by several years.

## Background

Olfactory hallucination is an uncommon type of hallucination in which the individual reports olfactory perceptions in the absence of chemical stimuli. Olfactory hallucination has been reported in 4.2–14.5% of the general population (1, 2). In more than half of affected individuals, olfactory hallucination occurs together with auditory, visual, or tactile hallucinations (2). Olfactory hallucinations, similar to other psychotic-like experiences, are more common in young individuals (< 40 years) with less education who have been exposed to alcohol and drugs or who have experienced stressful and traumatic events (2). Olfactory hallucinations have been observed in various neurological disorders, such as Alzheimer’s disease (3), Parkinson’s disease (4), epilepsy (5), and migraine (6), as well as in psychological disorders, such as schizophrenia (7), depression (8), bipolar disorder (9), and substance abuse (10). Changes in olfactory sensitivity have also been observed in patients with anorexia, post-traumatic stress disorder (11), bulimia (12), and autism (13). Olfactory hallucinations have been significantly associated with self-reported anxiety symptoms and stressful life events (14). Several studies have specifically indicated an increased sensitivity to internal and external sensory cues in patients with panic disorder (15, 16). The previous study also found that panic disorder patients appeared to be highly sensitive, reactive and aware of odors relative to healthy controls (17). The majority of research reported that anxiety disorders have detrimental effects on neuropsychological performance, such as executive function, memory, attention, and learning (18). People having health anxiety are mainly worried about and inaccurate interpret their body symptoms and might present psychotic-like symptoms, such as hypochondria (19). Furthermore, olfactory hallucinations may increase the likelihood of developing psychopathology: patients with olfactory hallucination develop symptoms of mood disorders whose severity correlates with severity of olfactory loss (2).

No consistent effective treatment strategies have been reported for olfactory disorders (20, 21), and such hallucinations have rarely been identified as the initial symptom of anxiety disorders. Here, we present a rare case of an elderly woman who experienced olfactory hallucination that worsened over a period of almost 4 years. After admission to our hospital, she was diagnosed with generalized anxiety disorder and treated with anti-anxiety medication, which effectively eliminated the olfactory hallucination.

## Case presentation

### Chief complaints

A 66-year-old Chinese woman was referred to our hospital in June 2021 due to olfactory hallucination. In 2004 the patient had been admitted to our hospital due to insomnia and nightmares. She had difficulty in initiating sleep and frequently awaked almost every night. The sleep disturbance caused her distress in daily life. She was diagnosed with insomnia disorder. Administration of 12.5 mg doxepin per night improved her sleep, and the patient reported stable mood and good social function during follow-up. However, in May 2017 she gradually manifested olfactory hallucinations, claiming that she could smell some Chinese medicines, even in wide open spaces on mountains. She did well in her job as a storekeeper and took good care of her grandson. At first, she went to the otorhinolaryngologic department, but the nasal endoscopy revealed normal structural nasal cavity or nasopharynx, axial and coronal computed tomography of the nose did not show abnormalities, so she did not get any treatment but psychiatric consultation was recommended. She was diagnosed with brief psychotic disorder after 20 days of olfactory hallucination onset in a local hospital, and she denied manic, depressive or anxious mood, denied unexpected panic attacks, denied psychotic symptoms except olfactory hallucination. The psychological tests administered by clinicians indicated a Hamilton Depression Scale (HAMD) score of 5, Hamilton Anxiety Scale (HAMA) score of 3. She received olanzapine (10 mg/night) for 2 months, followed by aripiprazole (20 mg/day) for 1 month, but her symptoms continued.

When the patient was admitted to our hospital in June 2021, she reported that 6 months before, her olfactory hallucinations had worsened and that the phantom smells had changed to something like a mixture of scallion, ginger, and garlic. At the same time, she reported being unable to smell normal scents, being unable to fall asleep at night, feeling restless and fatigued, having difficulty concentrating on daily activities, and often feeling limb muscle tension and sweating. She worried a lot about her daughter’s marriage, feared that some accidents would happen to her family members. She can still take care of her grandson. She denied depressed mood, unexpected panic attacks, and phobic avoidance.

### Personal history and family history

The patient had completed primary school and had worked as a storekeeper until her retirement. She reported having grown up in a strict family environment and having a bad relationship with her ex-husband. She brought up her daughter alone and she lives alone since her daughter got married.

### History of past illness

She had a history of tuberculosis and cholecystectomy. In 2011 she had been diagnosed with hyperlipidemia and since then she had been taking 5 mg atorvastatin every night. The patient and her family members denied any drug abuse, smoking or drinking. She had no family history of mental disorders.

### Physical examination and laboratory examinations

**FIGURE 1:**
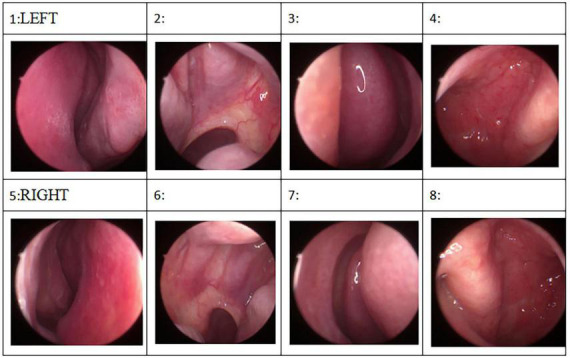
Endoscopy failed to reveal obvious structural auxiliary abnormalities, or abnormalities in the nasal cavity or nasopharynx.

**FIGURE 2:**
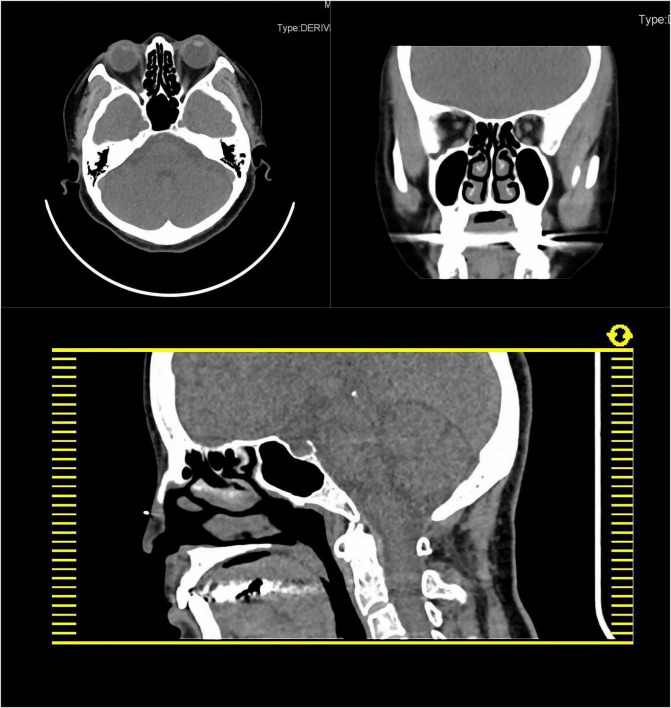
Axial and coronal computed tomography of the nose showed only paranasal sinusitis.

**FIGURE 3:**
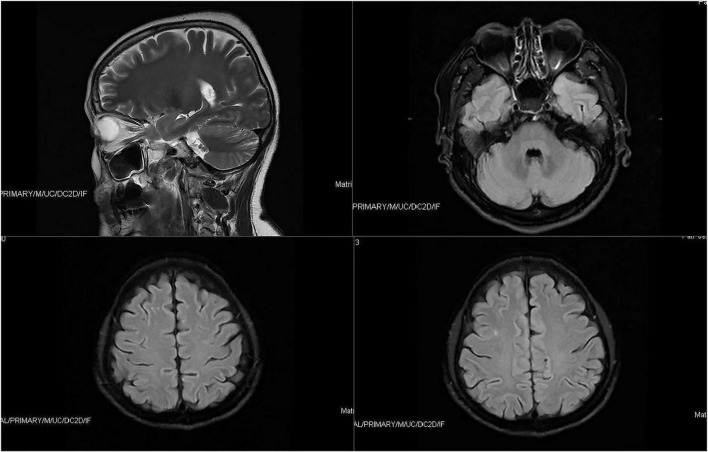
Brain magnetic resonance imaging only showed a few ischemic foci in the brain parenchyma and paranasal sinusitis.

In light of the patient’s long history of olfactory hallucination, the otorhinolaryngology department was asked to examine her, and those clinicians suggested the possibility of phantom olfactory perception. To exclude organic lesions, auxiliary examination and nasal endoscopy were performed. Endoscopy failed to reveal obvious structural auxiliary abnormalities, or abnormalities in the nasal cavity or nasopharynx (see Figure 1). Axial and coronal computed tomography of the nose showed only paranasal sinusitis (see Figure 2). And brain magnetic resonance imaging only showed a few ischemic foci in the brain parenchyma and paranasal sinusitis (see Figure 3). Chest computed tomography showed soft tissue nodules in the anterior basal segment of the lower lobe of the right lung, while both lungs showed multiple, scattered, small inflammatory nodules as well as mild chronic inflammation. Bilateral pleural thickening and adhesion, enlarged mediastinal lymph nodes, and aortic wall calcification were also observed. Thyroid color Doppler ultrasonography revealed bilateral lobular thyroid nodules suggestive of nodular goiter, while conventional color ultrasonography of the abdomen and urinary system indicated the presence of fatty liver, liver cyst, dilated common bile duct, and enlarged spleen. Electrocardiography and electroencephalography were unremarkable.

Neurological examination showed no obvious abnormalities, and her apolipoprotein E genotype was E3/E3, suggesting low risk of Alzheimer’s disease. In addition, no obvious abnormalities were found in routine blood or urine indices, liver or kidney function, electrolytes, blood lipids or glucose, coagulation or thyroid function, tumor markers, or glycosylated hemoglobin.

### Further diagnostic work-up

Assessment of symptoms was based on a Chinese version of HAMD and HAMA administered by a psychiatrist. HAMD score of 8, HAMA score of 22. Chinese version of Childhood Trauma Questionnaire score 33 (emotional abuse score 9, physical abuse score 5, sexual abuse score 5, emotional neglect score 9, and physical neglect score 5) which indicated without childhood trauma (22). Mini-mental State Examination score25, and Montreal Cognitive Assessment score of 24, indicating no obvious abnormalities in cognitive function.

### Final diagnosis

Based on the patient’s reported symptoms and the tests, the patient was diagnosed at our hospital with generalized anxiety disorder based on the Diagnostic and Statistical Manual of Mental Disorders, 5th Edition (23).

### Treatment

**FIGURE 4:**
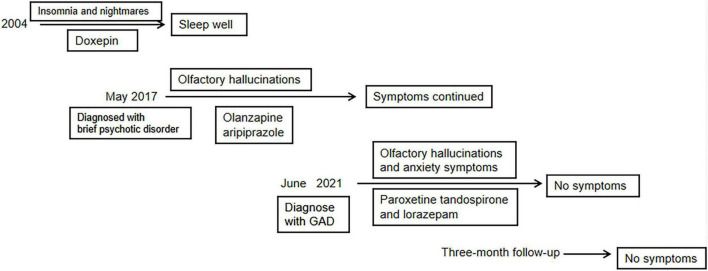
The timeline of treatment.

After diagnosis, the patient was treated with paroxetine (20 mg/day), tandospirone (10 mg three times a day), and lorazepam (0.5 mg/night) to relieve anxiety and control sleep disorders, as well as atorvastatin calcium (5 mg/night) to control hyperlipidemia. This medication was combined with electroencephalographic biofeedback training and repetitive transcranial magnetic stimulation once a day. After 4 days of this therapy, the patient started to smell strong odor of ammonia in the restroom, especially in the afternoon. Therefore, we adjusted the medication to tandospirone (10 mg three times a day), paroxetine (40 mg/day), and lorazepam (0.25 mg/noon and 0.5 mg/night). After 6 days on the modified drug regime, the patient claimed that the odor became mild, but she still had hyposmia for normal smells, especially in the afternoon. After 10 days on the physiotherapy and modified drug regime, the patient reported feeling relaxed and sleeping well at night, with no olfactory hallucination. After a total of 20 days hospitalization, transcranial magnetic stimulation sessions were stopped. She was discharged on tandospirone at 10 mg three times a day, paroxetine at 40 mg/day, and lorazepam 0.5 mg/night. She scored 7 on the HAMD and 8 on the HAMA. A timeline of treatment is shown in Figure 4.

### Outcome and follow-up

At 3-month follow-up, she reported no olfactory hallucination, and she slept well. She scored 5 on the HAMD and 4 on the HAMA. She lived with her daughter and took good care of her grandson every day. She denied anxious and her mood was relatively easy to maintain a smooth. She also had good tolerability of the medication.

## Discussion and conclusion

Olfactory dysfunction can be quantitative or qualitative, and the latter type is also known as olfactory hallucination (phantosmia) or parosmia (troposmia) (1). Olfactory dysfunction has an obvious negative impact on mental health, social skills, relationships, wellbeing, and life quality (24), yet most affected individuals are unaware of the disorder. The majority of the research reported that anxiety disorders have detrimental effects on neuropsychological performance, such as executive function, memory, attention, and learning, which adversely affecting patients’ lives (18). Several diseases have been shown to impair olfactory function, such as upper respiratory infection, nasal and paranasal sinus disease, head trauma, neurodegenerative disease, epilepsy, and psychiatric disorders (25). However, we are unaware of reports that olfactory dysfunction can be the first symptom of anxiety disorder. Our case indicates that such hallucination may be a prodromal symptom in patients with anxiety disorder, and that it can be effectively treated with anti-anxiety medication.

The previous studies suggest that paranasal sinusitis may cause olfactory hallucinations (26). But the nasal endoscopy and the axial and coronal computed tomography of the nose did not show any abnormity when the first-time olfactory appeared in 2017, and this time we did not do any targeted treatment about paranasal sinusitis, and after the only antianxiety therapy, the odor disappeared. So, the olfactory hallucination may not be caused by paranasal sinusitis.

Since smell dysfunction is one of the first signs of neurodegenerative diseases (27), we also performed neurological examinations. The patient did not show characteristic Alzheimer’s symptoms such as ecmnesia, or characteristic Parkinson’s symptoms such as static tremor, bradykinesia, muscle rigidity, or abnormal posture or gait. In addition, only a few ischemic foci were detected in the brain parenchyma and paranasal sinusitis, and the patient showed a E3/E3 genotype for apolipoprotein E, indicating low risk of Alzheimer’s disease. Furthermore, no abnormalities were observed by electroencephalography, and the patient denied any seizures, allowing us to exclude neurodegenerative disease as a cause of the olfactory hallucination.

The patient’s symptoms led us to examine whether she might have anxiety disorder. Olfactory dysfunction is a marker for depression (8) and it may be associated with schizophrenia (7), bipolar disorder (9), anorexia (12), bulimia (12), post-traumatic stress disorder (11), and autism (13). Indeed, low scores on olfactory tests have been found useful for early diagnosis of such diseases (28). Our patient had refused to take olfactory tests in the past, and she again refused at admission to our hospital. She also denied other psychotic symptoms, such as delusion and abnormal behavior, and her earlier treatment with antipsychotics was ineffective. She claimed no depression or elevation in mood at admission to our hospital, she reported symptoms consistent with anxiety syndrome, and her HAMA score was 22, confirming the presence of generalized anxiety disorder.

Earlier epidemiologic studies have shown that olfactory dysfunction and anxiety occur more often in women younger than 40 years (29) who have less education and poor mental health, who have been exposed to alcohol and drugs, and/or who have experienced stressful and traumatic events (2). Although our patient was older and was not taking alcohol or drugs when olfactory hallucination appeared, she was still clearly suffering from anxiety, highlighting the need to pay attention to anxiety disorder in the elderly.

The association of olfactory dysfunction with anxiety disorder could be attributed to negative life experiences. Our patient reported having grown up in a strict family and an unpleasant childhood but without childhood trauma. She also reported having a bad marriage, and a stressful everyday life. These chronic stress may have led to changes in neural circuits, including the olfactory system, as already reported for animal models and humans (30). Moreover, anxiety and olfaction may have common cerebral substrates. Earlier studies have reported a close relationship between olfactory and emotional processing, as they share a common brain pathway (31). The hippocampus (32) and amygdala (33) in the primary olfactory cortex, as well as the insula (34) and the orbitofrontal cortex (35) in the secondary olfactory cortices, may play key roles in encoding, learning, and regulating emotions, especially negative emotions such as sadness, unhappiness, and rage (33). Functional magnetic resonance imaging of patients with generalized anxiety disorder has confirmed the involvement of the prefrontal lobe and amygdala in disease pathogenesis (36), while abnormal activation of the amygdala and the orbitofrontal region has been observed in mood disorder (37). Thus, generalized anxiety disorder and olfactory disorders may have the same neuropathogenesis involving lesions in the prefrontal lobe and amygdala. In fact, anxiety patients have shown deficits in odor discrimination and altered odor perception (38), suggesting that olfactory hallucination may be a symptom of anxiety disorder.

There is evidence of the increased olfactory sensitivity among anxiety disorders and even the association with joint hypermobility syndrome (JHS), which is a benign heritable collagen condition that is featured by increased laxity of the joints, resulting in enhanced distensibility in passive movements and hypermobility in active movements of joint (39, 40). The previous study found that suffering from JHS in patients with panic disorder showed higher odor acuity, greater reactivity to smells and also increased odor awareness (16). A 15-year follow-up study indicated that JHS was a risk factor in the development of panic disorder, highlighting the importance of assessing JHS among patients with anxiety disorders (41). Individuals suffering from JHS frequently report symptoms associated with autonomic nervous system abnormalities and stress-sensitive illnesses (39, 42). Although our patient did not present the obvious abnormalities of joint movements and autonomic nervous system, such as syncope, fatigue, chest discomfort and orthostatic hypotension, it suggested that a detail physical examination by using the Hospital del Mar criteria to assess JHS symptoms in patients with anxiety disorder was required (43). Phantosmia has been clinically related to schizophrenia and mood disorders: these mental diseases and perception of phantom smells have been linked to abnormal levels of several neurotransmitters such as acetylcholine, dopamine, and norepinephrine (27). It has been difficult to isolate the effects of each neurotransmitter on olfactory hallucination because affected patients show cell losses within the cholinergic nucleus basalis of Meynert, the noradrenergic locus coeruleus, the serotonergic raphe nuclei, and the dopaminergic ventral tegmental area, suggesting that all these neurotransmitters interact with one another (27). Abnormal levels of 5-hydroxytryptamine and norepinephrine have also been found in patients with anxiety (44), suggesting further implicating neurotransmitters in the link between phantom smells and anxiety disorder.

Clinical treatment of olfactory disorders has not yet been standardized (20, 21). Our patient showed no inflammation or head injury, and her treatment with antipsychotic drugs at a local hospital had no effect on olfactory hallucination. Considering the patient’s severe anxiety symptoms, we provided her with anti-anxiety medication, which completely eliminated the olfactory hallucination. This experience indicates that anti-anxiety drugs may treat this symptom effectively in patients with generalized anxiety disorder.

Assessing a patient’s perspective on treatment is part of an integrated approach to treatment optimization (45, 46). The previous study also found that seeking help for emotional concerns is challenging due to stigma and unfamiliar symptoms (47). High psychological distress was associated with low satisfaction with provider–patient interactions and the presence of an anxiety disorder was associated with low satisfaction in adequacy of care (48). Our patient did not know where to get help and how to cope with the olfactory hallucination. She prefers to hear positive information from other people who have experienced the same symptom. However, she felt distressed when she heard neurodegenerative diseases or psychotic disorder maybe suffered. For this treatment, we provided information about the nature and causes of anxiety disorder and olfactory hallucination, medication, side effects and how to cope with the daily problems. She was satisfied with the treatment and had good compliance. The previous study also suggested the need for increased attention when delivering care to older adults with mental health problems (48).

## Conclusion

In summary, our findings clearly support that olfactory hallucination can be the first and only symptom of anxiety disorder, preceding diagnosis of the disorder by several years, even in elderly individuals who have experienced stressful life events but have never consumed drugs or alcohol. We also found that anti-anxiety medication maybe an effective approach to treating olfactory hallucination in such patients. Further studies should investigate the mechanisms underlying the association of olfactory hallucination with anxiety.

## Data availability statement

The original contributions presented in this study are included in the article/supplementary material, further inquiries can be directed to the corresponding author.

## Ethics statement

Written informed consent was obtained from the individual(s) for the publication of any potentially identifiable images or data included in this article.

## Author contributions

XJ was the major contributor in writing and revising the manuscript. YY and ZXL interpreted the patient data. YO collected the patient data. ZL made the substantial contribution to the conception and design of the work. All authors read and approved the final manuscript.

## Abbreviations

HAMD: Hamilton Depression Scale
HAMA: Hamilton Anxiety Scale.
